# Surface Roughness, Dynamic Wettability, and Interphase of Modified Melamine Formaldehyde-Based Adhesives on Jabon Wood

**DOI:** 10.3390/polym16081084

**Published:** 2024-04-12

**Authors:** Yusup Amin, Naresworo Nugroho, Effendi Tri Bahtiar, Wahyu Dwianto, Muhammad Adly Rahandi Lubis, Ulfa Adzkia, Lina Karlinasari

**Affiliations:** 1 Research Center for Biomass and Bioproducts, National Research and Innovation Agency, Cibinong 16911, Indonesia; wahy002@brin.go.id (W.D.); muha142@brin.go.id (M.A.R.L.); 2 Department of Forest Products, Faculty of Forestry and Environment, IPB University, Bogor 16680, Indonesia; nares@apps.ipb.ac.id (N.N.); bahtiar_et@apps.ipb.ac.id (E.T.B.); ulfaadzkia@apps.ipb.ac.id (U.A.)

**Keywords:** contact angle analysis, dynamic mechanical analysis, melamine formaldehyde, Jabon wood, wood–adhesive interphase

## Abstract

The surface roughness and wettability of wood are critical aspects to consider when producing laminated wood products with adhesive applications. This study aims to investigate the surface roughness and dynamic wettability of Jabon wood in the presence of melamine formaldehyde (MF)-based adhesives. Commercial MF adhesives (MF-0) and modified MF adhesives (MF-1) were applied to Jabon wood, which includes tangential (T), radial (R), and semi-radial (T/R) surfaces. The surface roughness of Jabon wood was assessed using a portable stylus-type profilometer. The low-bond axisymmetric drop shape analysis (LB-ADSA) method was employed to identify the contact angle (*θ*) of the MF-based adhesives on Jabon wood. The wettability was determined by evaluating the constant contact angle change rate (*K* value) using the Shi and Gardner (S/G) model. Dynamic mechanical analysis (DMA) was employed to investigate the viscoelastic characteristics of the interphase analysis of the wood and MF-based adhesives. The roughness level (Ra) of the Jabon board ranged from 5.62 to 6.94 µm, with the T/R having a higher level of roughness than the R and T. MF-0 exhibited a higher *K* value (0.262–0.331) than MF-1 (0.136–0.212), indicating that MF-0 wets the surface of Jabon wood more easily than MF-1. The wood–MF-0 interphase reached a maximum stiffness of 957 N/m at 123.0 °C, while the wood–MF-1 had a maximum stiffness of 2734 N/m at 110.5 °C. In addition, the wood–MF-0 had a maximum storage modulus of 12,650 MPa at a temperature of 128.9 °C, while the wood–MF-1 had a maximum storage modulus of 22,950 MPa at 113.5 °C.

## 1. Introduction

Adhesive quality is a critical component that must be meticulously evaluated during the production of laminated timber products [1]. In the manufacturing of wood products, surface preparation is a critical step preceding the gluing or finishing procedures [2,3,4,5]. Sanding is a method that is frequently employed in this process. The wettability of wood is directly influenced by the texture of its surface, which is itself altered by sanding [6,7]. The properties of the liquid being applied and the characteristics of the wood itself are the two primary determinants of liquid penetration into wood [8,9]. Furthermore, Walinder (2000) [9] states that investigations into wetting phenomena on wood surfaces could contributefundamental knowledge regarding a wood material and its intricacies, in addition to being essential for comprehending the interaction between wood and substances such as adhesives or coatings. Wetting refers to the phenomenon that occurs when a liquid makes contact with a solid surface [10,11]. In other terms, wetting is an intermolecular interaction that occurs when liquids and solids come into direct contact at their interfaces [9]. So, the roughness and molecular physicochemical properties of solid surfaces reatlyaffect wetting phenomena and the transmission of liquids [12,13].

The penetration of a liquid into wood pores is a crucial aspect of bond formation since wood is a porous material [10]. The principles of adhesive wetting on a wood surface include the creation of a contact angle at the solid–adhesive interface, the spread of the adhesive over a solid surface, and the penetration of the adhesive into the porous solid substrate [10,14]. In several recent investigations [3,11,15,16,17,18,19,20], the wettability qualities of wood were determined by measuring the contact angle between a liquid and the surface of the wood. As an anisotropic material, the adhesive wetting properties of wood may be different along and across the wood grain direction [8,10]. Numerous factors influence wood wettability, including surface roughness and machining conditions [14,21], wood species and the location of wood [10,22], and treatment and drying methods [23]. In addition, Yuan and Lee [24] have reported that the factors of wood characteristics and a liquid’s properties affect wettability.

Numerous adhesives can be used to fabricate laminated wood products such as urea formaldehyde (UF), phenol formaldehyde (PF), melamine formaldehyde (MF), melamine–urea formaldehyde (MUF), emulsion polymer isocyanate (EPI), and polyurethane [25,26,27,28,29]. Among them, MF adhesives are one type of adhesives ordinarily used to produce laminated wood products [1,26,27,29], such as cross-laminated timber (CLT), glued laminated timber (GLT), and laminated veneer lumber (LVL). Alongside PF adhesives [25], MF adhesives are extensively used in the production of semi-exterior- and exterior-grade wood-based panels [29]. These adhesives are widely selected by glulam or CLT producers because of their relatively low costs, transparent bonding lines, excellent durability, heat resistance, and resistance to water and moisture [30,31]. MF, being a thermosetting glue, is typically utilized in conjunction with hot presses, resulting in higher costs associated with heat energy consumption. Modifying formaldehyde-based adhesives is a remarkably effective strategy for increasing the economic advantages of these adhesives and preserving the environment [25]. A recent innovation concerning the modification of MF-based adhesives for CLT production from Jabon wood by means of cold-pressing application has been successfully developed [1]. Nevertheless, the specific attributes of the bonding mechanism between Jabon wood and MF-based adhesives have yet to be discovered. The bonding quality of a laminated wood product is influenced by the surface roughness and wettability qualities of the wood. This study aims to investigate the surface roughness and wettability of Jabon wood (*Anthocephalus cadamba* Roxb. Miq.) in the presence of an MF-based adhesive.

## 2. Materials and Methods

### 2.1. Materials

The two main materials used in this study are Jabon wood and MF resin as an adhesive. Jabon wood (*Anthocephalus cadamba* Roxb. Miq.) with a density of 0.44 g/cm^3^ and a moisture content of 12 ± 2% was obtained from a community forest located in the Bogor Region, West Java. Meanwhile, the commercial melamine adhesive (MA-204) was supplied by PT Pamolite Adhesive Industry, Probolinggo, East Java, Indonesia; Polymeric 4,4-methylene diphenyl diisocyanate (pMDI) was supplied by PT Anugerah Raya Kencana, Tangerang, Banten, Indonesia. Additional materials such as wheat flour and technical-grade citric acid powder were either obtained from a commercial market or provided by the Integrated Laboratory of Bioproducts (iLab), National Research and Innovation Agency (BRIN), Cibinong, Indonesia.

### 2.2. Methods

#### 2.2.1. Surface Roughness Test

**Figure 1:**
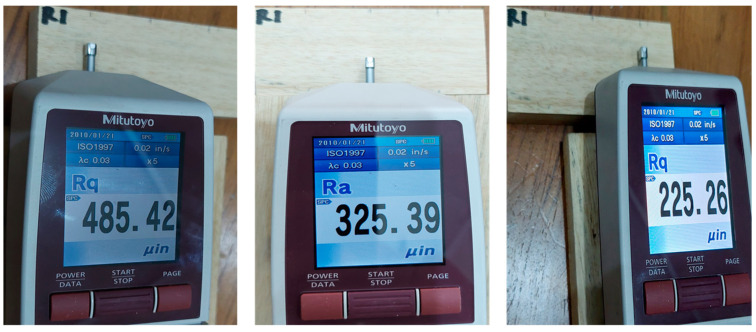
The surface roughness measurement of Jabon wood specimens in three different positions using a portable stylus-type profilometer (Mitutoyo Surftest^®^ SJ-210).

Jabon lumbers with a dimension of 90 mm × 40 mm × 15 mm in length, width, and thickness were used for measuring surface roughness. In conformance with ISO 1997, the surface roughness of Jabon wood lumbers was determined using a portable stylus-type profilometer (Mitutoyo Surftest^®^ SJ-210, Mitutoyo Corporation, Kanagawa, Japan), after sanding with a 100-grit belt sander (P100). The surface roughness measurement of the wood specimens was performed perpendicular to the fiber direction in three different positions (Figure 1) by a diamond tip with a radius of 5 mm, a tracing length of 6 mm, a cut off of 0.8 mm, and a speed of 0.5 mm/s [3,15,32]. The arithmetical mean roughness value (Ra) was used to assess the surface roughness level [3,4,11,14,15]. Ra measurements were conducted on tangential (T), radial (R), and semi-radial (T/R) cross-sections of Jabon lumbers, with each kind of lumber being tested over three replicas. The Keyence VHX 6000 Digital Microscope (Keyence Corporation, Osaka, Japan) was used for observing the surface topography profile of Jabon wood on three different surfaces. This microscope can generate high-quality digital images [33]. The ultrasmall zoom lens models VH-Z20T and VH-250T dual-light enable high-magnification recording, with magnification capabilities of 20–200× and 250–2500×, respectively.

#### 2.2.2. Contact Angle Measurement

Commercial melamine formaldehyde adhesives (MF-0) and modified MF adhesives (MF-1) were chosen to investigate the wettability of adhesives on Jabon wood. In this research, MF-1 was composed, starting from MF-0, by adding citric acid (20 wt%) as a catalyst, pMDI (96.2 wt%) as a cross-linker, and wheat flour as a filler [1]. Citric acid and pMDI were added based on the solids content of the control adhesive: 5% and 3%, respectively. Wheat flour made up as much as 10% of the total mixture. The resulting mixture was then manually stirred for 1–2 min at 27 ± 2 °C, yielding a cold-setting melamine-based adhesive. According to a previously published study, the fundamental properties of the adhesives used in this study are shown in Table 1 [1].

The evaluation of wettability was performed through the measurement of the dynamic contact angle between the MF-based adhesives and the surface of Jabon wood. Distilled water (aquades) was employed as a control. Each of these liquids, i.e., MF-0, MF-1, and aquades, were dripped using a micropipette and a syringe onto the Jabon wood surface, employing the same specimen and point location as the surface roughness test. The sessile droplet volume used for the experiment was 0.02 mL for aquades [3,32] and about 0.03 mL for the MF-based adhesives [11,15].

**Figure 2:**
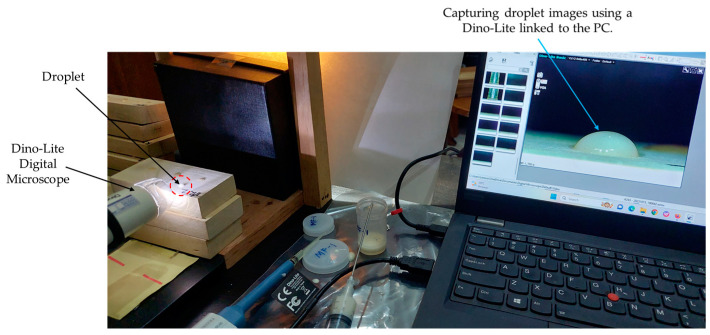
Capturing droplet distribution and change patterns with a Dino-Lite Digital Microscope (Am 2111 series) adjusted for 40× magnification.

**Figure 3:**
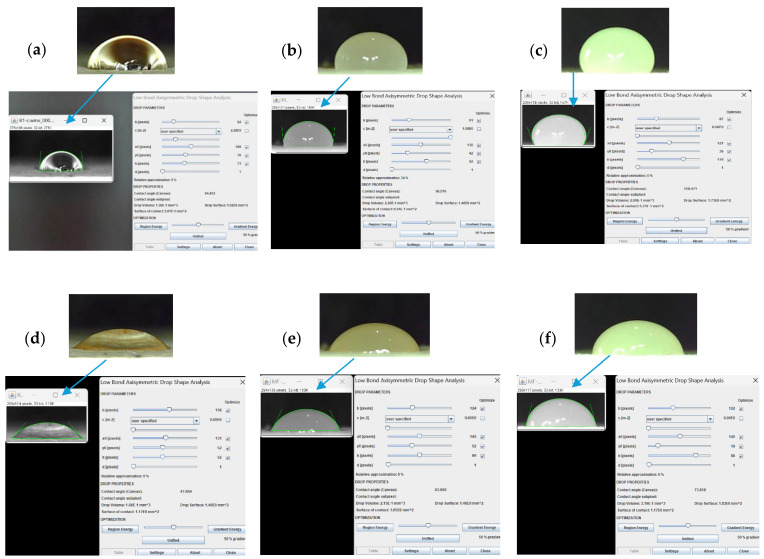
Contact angle measurements by LB-ADSA method: (**a**) aquades at 0 s; (**b**) MF-0 adhesive at 0 s; (**c**) MF-1 adhesive at 0 s; (**d**) aquades at 3 s; (**e**) MF-0 adhesive at 3 s; and (**f**) MF-1 adhesive at 3 s.

The experiment involved using a Dino-Lite Digital Microscope Basic Am 2111 series (AnMo Electronics Corporation, New Taipei City, Taiwan) with a USB camera connected to a personal computer (PC) to record videos (Figure 2). A Dino-Capture tool with a magnification of 40× was utilized to record the process of droplet distribution and absorption from the initial placing until the water penetrated the wood surface. The video data in a Windows media video (WMV) format were further processed using the GOM Player software (GOM Player 2.3.90.5360) to extract visual segments at intervals of 3 s, resulting in seven individual images, for a total duration of 18 s. As in the surface roughness test, the contact angle measurements were conducted on T, R, and T/R surfaces of Jabon wood in three replicas, respectively. The contact angle (θ) of an individual drop image was determined using the low-bond axisymmetric drop shape analysis (LB-ADSA) method, provided by the Image-J software (ImageJ 1.46r) [34]. In contrast to predominant droplet shape analyses, such as the drop-snakes analysis method [3,4,8], the LB-ADSA method fails to distinguish between left and right droplet image contours and contact angles. The approach applied by LB-ADSA is that the contact angles on the left and right sides of an object are identical (Figure 3), resulting in a reflection resembling a mirror on both sides [34].

#### 2.2.3. Determination of Equilibrium Contact Angle and Constant Contact Angle Change Rate

When the liquid was first poured on the wood surface, the droplet was normally circular and gradually flattened over time [15]. With time, the drop shape tended to stabilize, resulting in an equilibrium contact angle (*θ*_e_). The change in the contact angle as a function of time was determined using a segmented regression model between the time (x) and the contact angle (y), using the SAS STAT’s PROC NLIN [3,11,15]. The rate of contact angle change is proportional to the rate of liquid penetration and spread across a solid surface [10,11]. In this study, wettability was quantitatively assessed by examining the constant contact angle change rate (*K* value) on the S/G model [10]. The *K* value indicates the rate at which a liquid distributes and permeates into the porous structure of wood. Increasing values of K correspond to a reduced time needed for the contact angle to reach a state of relative equilibrium and for the liquid to spread and permeate. The S/G model is explained by Equation (1):

(1) $$\theta =\frac{\theta_{i}\times \theta_{e}}{\theta_{i}+\left(\theta_{e}-\theta_{i}\right)\exp\left[K\;\left(\frac{\theta_{e}}{\theta_{e}-\theta_{i}\;}\right)t\right]}$$

where *θ* represents the contact angle at a certain time, *θ*_i_ is the initial contact angle, *θ*_e_ is equilibrium contact angle, *t* is the wetting time, and *K* represents the constant contact angle change rate. A non-linear regression model was used to calculate the K-value using the defined function to fit the S/G equation via XLSTAT Addinsoft (XLSTAT 2014.5.03) [3,11].

#### 2.2.4. Wood–Adhesives Interphase Analysis Using Dynamic Mechanical Analyzer

Dynamic mechanical analysis (DMA) can be employed to investigate the viscoelastic characteristics of the interphase analysis of wood and adhesives. Most polymeric materials or polymeric interfacial phases exhibit a viscoelastic behavior, which combines the characteristics of both solids (elasticity) and liquids (viscosity) [35,36,37]. Dynamic mechanical analysis (DMA 8000, Perkin Elmer Inc., Waltham, MA, USA) was utilized to investigate the MF resin and Jabon wood interphase. Each MF adhesive was used to bond two thin Jabon wood veneers (50 mm × 10 mm × 0.5 mm) with a glue spread of 300 g/m^2^. All the specimens were precured in an oven at 50 °C for 10 min before the DMA analysis. The storage modulus (E′), loss modulus (E″), and tan delta of each specimen were determined at a frequency of 1 Hz, a strain level of 0.01%, and a heating rate of 1 °C/minin the scanning range of 25–300 °C in the dual-cantilever mode [38].

#### 2.2.5. Mechanical Properties of Wood–MF Composites

Block shear strength samples were prepared by gluing Jabon wood with MF-0 and MF-1 resins. Two-ply composites were made via cold-pressing at 1 MPa for 2 h at different glue spreads of 250, 280, and 300 g/m^2^. Furthermore, the composites were conditioned for a week prior to block shear testing. A block shear strength analysis was undertaken to determine the bonding strength. The block shear samples were tested using a 50 kN universal testing machine (UTM AG-IS 50 kN, Shimadzu, Kyoto, Japan) with a crosshead speed of 2 mm/min.

## 3. Results and Discussion

### 3.1. Surface Roughness of Jabon Wood

The result in Table 2 shows the surface roughness value of Jabon wood after sanding with a 100-grit belt sander (P-100) on different surface types, determined using a portable stylus-type profilometer. The surface roughness of solid materials is generally measured using three parameters: average surface roughness (Ra), root mean square roughness (Rq), and ten-point mean roughness (Rz) [39]. However, Ra is the most used metric for determining wood surface roughness. Consequently, the Ra parameter was employed in this investigation to quantify the surface irregularity of Jabon wood. An approach methodology utilizing Ra values has been implemented in prior studies [3,4,5,14,15,39,40]. The average roughness (Ra) of a profile is calculated by averaging the individual depths and heights (irregularities) of its arithmetic mean elevation. In addition, Ra represents the mean deviation of the profile from the mean line, determined across the entire length of the assessment [41]. Jabon wood treated with a P-100-grit sander had an Ra of 5.62 µm on the tangential surface (T), 5.77 µm on the radial surface (R), and 6.94 µm on the semi-radial surface (T/R). The tangential surface had a lower Ra value than the radial or semi-radial surfaces, indicating that the roughness level of Jabon wood on a tangential surface was better than that of the radial and semi-radial surfaces. The findings of this study aligned with prior studies [41] indicating that the radial surface of rubberwood exhibited a rougher appearance compared to its tangential surface. The average Ra value of the three types of Jabon wood surfaces investigated in this study (6.11 µm) is comparable to prior research [15], which found that the average Ra value of Jabon wood before heat treatment was 6.22 m.

**Figure 4:**
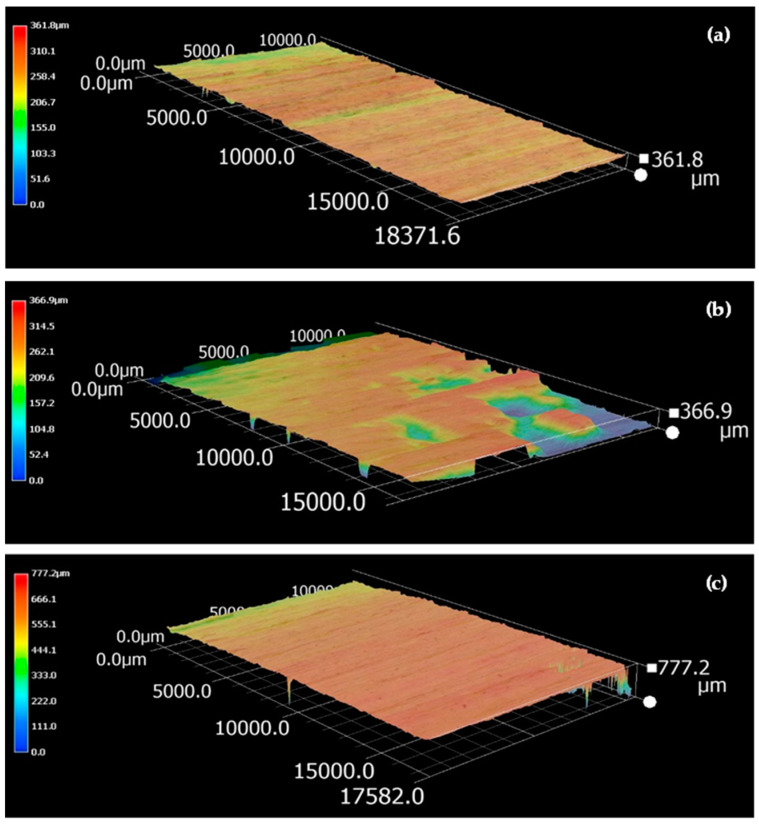
Three-dimensional surface topography profile of Jabon wood after being sanded with a 100-grit belt sander (P100) at a 100× magnification: (**a**) tangential surface; (**b**) radial surface; and (**c**) semi-radial surface.

The evaluation of surface quality can be accomplished through the utilization of topographic measurements [42]. Figure 4 shows the 3D surface topographical profile of Jabon wood after it was sanded with a 100-grit belt sander (P100) and analyzed with a digital microscope (Keyence VHX 6000, Keyence Corporation, Osaka, Japan). The tangential surface of Jabon wood (Figure 4a) had a lower maximum peak height value than the radial (Figure 4b) and semi-radial (Figure 4c) surfaces. This finding is comparable with the results obtained using the stylus method mentioned previously and in line with the results of previous studies [43]. Visually, Figure 4b,c demonstrate that the radial surface has greater amounts of pattern (color) changes than the semi-radial surface, rendering it rougher. However, it is crucial to highlight that significant fluctuations in the surface roughness profile do not imply a rougher surface when compared with a surface roughness profile with slight variations [43]. Based on a 3D analysis of the surface topographical profiles, the maximum value of the peak height has a greater impact on wood surface roughness than the frequency of color change patterns.

The surface properties of wood are essential in the manufacturing processes of wood products, such as adhesive bonding or finishing [5,41]. Wetting analyses, topography measurements, and cell damage evaluations can all help to determine surface quality. Numerous processing techniques used on wood can influence the structure, morphology, and chemical composition of the wood surface, hence altering the ability of liquids to wet the wood [14]. Different wood machining techniques result in various levels of liquid wettability on wood. Previous research [21] investigated the effect of surface conditions caused by various machining methods on the wettability properties of Mediterranean wood species. The same authors reported that sanded surfaces exhibited a significantly higher wettability compared to planed or disc-sawn surfaces. Sanding is a necessary and time-consuming procedure in the woodworking industry [6]. Sanded wooden surfaces are often varnished or glued in the manufacturing of furniture products. Several studies have been conducted to investigate the effect of sander grit size on the level of roughness of a wood surface [4,7,11,41]. Generally, the surface roughness of wood decreases as the grit number of the abrasive paper increases [11,41]. The sanding technique, whether applied tangentially, radially, or semi-radially, serves to refine the surface cells and make them smoother. The sanded surface disintegrates, creating fine particles (dust) which will occupy the pores on the surface [41]. A wood structure reveals variations in its constituents across its tangential, radial, and longitudinal surfaces [8]. An example of this distinction is the variation in the orientation of ray cells. The tangential surface is considerably smoother (lower Ra) than the radial surface. Possibly, the radial surface presents a greater obstacle during measurements than the tangential surface; this indicates that the radial surface is considerably coarser in texture [41]. It has been reported in other studies that surface roughness does not invariably diminish as sandpaper grain size increases [4]. Wood surface roughness parameters do not indicate that sanding with an abrasive of a smaller grain size results in a smoother surface [7]. Therefore, in our study, it was determined that 100-grain sandpaper (P-100) would be adequate to produce Jabon lumber with a suitable surface for applying MF-based adhesives in the manufacturing of laminated wood products. Previous investigations [44,45] verified a substantial rise in surface roughness parameters when P-100-grit sandpaper was utilized. The abrasive grains formed deep grooves, peak heights, and cell wall fibrillation on the wood surface, accelerating the dispersion of liquid on said surface. In line with Dai et al. (2019) [46], Niaraki and Krause [47] reported that the wood surfaces developed a larger number of interface areas after sanding. Consequently, adhesives are capable of more readily penetrating a wood’s depths and dispersing more uniformly across its surface.

### 3.2. Contact Angle and Dynamic Wettability

The capability of a liquid to generate a contact interface with a solid surface is referred to as wetting [48]. The wetting characteristics of wood are classified based on the contact angle (*θ*) formed between liquid droplets and the surface of the wood [11,15,48,49]. The roughness of a wood surface is directly linked to its wettability. A higher surface roughness corresponds to a greater surface hydrophilicity, resulting in more effective wetting and a lower contact angle [3,11,24,50].

**Figure 5:**
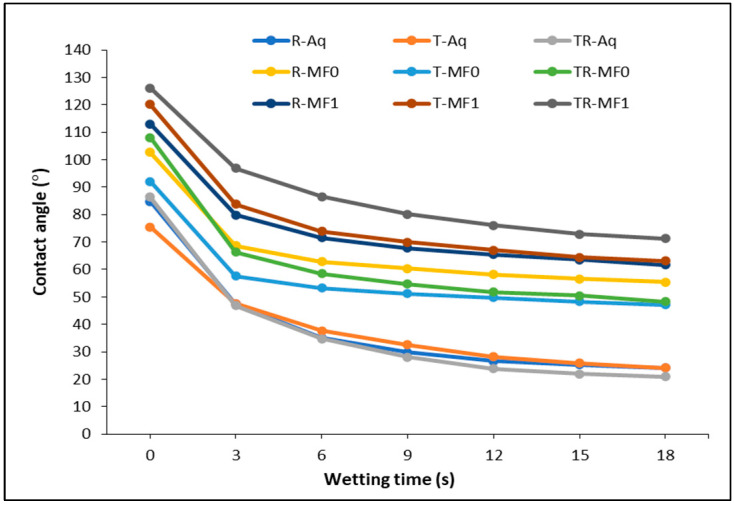
Contact angle changes as a function of time for aquades (Aq), MF-0, and MF-1 on different Jabon wood surfaces.

The wettability of wood has a role in the construction of an adhesive system. The wettability of the aquades and MF-based adhesives applied to various Jabon wood surfaces in our study could be quantitatively investigated with the S/G model [10,11]. Table 3 shows the initial contact angles (*θ*_i_), equilibrium contact angles (*θ*_e_), contact angle reduction ratio, *K* value, and R squared values for all the wood surfaces and liquid treatments examined. The change in contact angle over time during the liquid absorption process is a reducing function [10]. Liquid penetration and spreading occur in conjunction with the formation of a contact angle at the wood surface when a liquid drop is applied on it. In the early phases, until the third minute of the wetting process, there is a rapid decrease in the contact angle of a liquid drop (Figure 5). The aquades treatment exhibits the highest average contact angle reduction ratio (64.35–72.16%) compared to MF-0 and MF-1. The contact angle reduction ratio of MF-0 (42.83–51.18%) is slightly higher than that of MF-1 (41.21–44.55%). The effect angle gradually diminishes over time, ultimately achieving a state of relative equilibrium [10]. As shown in Table 3 and Figure 5, generally, adhesives based on MF exhibit higher initial contact angles and equilibrium contact angles than those of aquades. In the aquades treatment, the equilibrium contact angle is reached after 8.46–11.13 s. Meanwhile, the treatments MF-0 and MF-1 take 5.25–5.95 and 7.80–11.50 s, respectively, to achieve the equilibrium contact angle. In addition, according to [51], an equilibrium contact angle is achieved on a surface when an adhesive’s adhesion is counterbalanced by the solid surface’s tension over time. The time required to achieve the equilibrium contact angle is impacted by various parameters, such as the initial contact angle, the contact angle reduction ratio, and the properties of the liquid employed. When addressing aquades or MF-based adhesives, the time required to reach the contact angle tends to be longer, as the initial contact angle increases and the contact angle reduction ratio decreases. By treating aquades, decreasing the time required to reach the equilibrium contact angle tends to enhance the *K* value. This indicates that the wood surface becomes easily wet [4,11,52]. During the MF-based adhesive treatments, MF-1 takes more time to reach the equilibrium contact angle compared to MF-0. The viscosity difference between the MF-0 and MF-I adhesives also exhibits an effect, alongside the initial contact angle and contact angle reduction ratio. The higher viscosity of MF-1 compared to MF-0 [1] hinders the absorption of the adhesive into the wood surface, resulting in a longer time required to reach the equilibrium contact angle.

The contact angle of the modified MF (MF-1) is higher than that of MF-0, which is commercially available. A high wettability can be determined by contact angles below 90 degrees, which signifies that the liquid efficiently wets the wood surface. A contact angle exceeding 90 degrees signifies an insufficient wettability, wherein the liquid fails to sufficiently lubricate the wood surface [4,11,53]. Previous studies [40,54] reported that an increase in surface roughness results in a decrease in the contact angle, which subsequently leads to enhanced wettability and bonding performance. According to the findings of the current study and the above theory [40,54], the MF-0 adhesive has a higher bonding performance than MF-1 because its contact angle is lower. A higher *K* value indicates a more wettable surface [11]. Overall, Table 3 shows that the MF-0 adhesive exhibits a higher *K* value (0.262–0.331) than the MF-1 adhesive (0.136–0.212), indicating that the MF-0 adhesive wets the surface of Jabon wood more easily than the MF-1 adhesive. This result also shows that the wetting model had R squared values greater than 0.904 across all the wood samples examined in our study. Consequently, the S/G wetting model could be used to precisely characterize the hydration process of MF-based adhesives on Jabon wood surfaces. Nevertheless, prior research [1] shows that the MF-1 adhesive exhibits a superior bonding performance in terms of block shear strength and delamination compared to MF-0. The observed phenomenon can be attributed to the higher viscosity of MF-1 in comparison to MF-0 (Table 1). This viscosity factor restricts the absorption of the MF-1 adhesive into the wood surface, resulting in a greater contact angle of MF-1 compared to MF-0. There are other factors, in addition to the contact angle and the wettability of the wood surface, that impact the performance of wood bonding when a particular type of adhesive is utilized.

**Figure 6:**
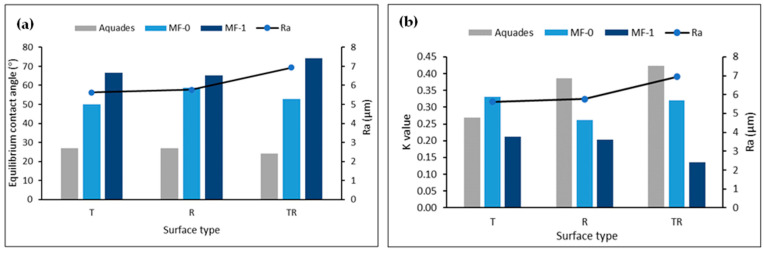
(**a**) Equilibrium contact angle for aquades, MF-0, MF-1, and Ra value on different Jabon wood surfaces, and (**b**) the *K* value for aquades, MF-0, MF-1, and Ra value on different Jabon wood surfaces.

Figure 6a,b show how changes in the surface roughness of Jabon wood affect the equilibrium contact angle and wettability of MF-based adhesives. In general, the surface roughness variances between Jabon wood’s three surface areas did not have much of an impact on the equilibrium contact angle. The equilibrium contact angle decreased slightly after the aquades treatment as roughness increased on the radial and semi-radial surfaces. Meanwhile, in the MF-0 and MF-1 treatments, the equilibrium contact angle fluctuated due to the increased surface roughness of Jabon wood. As the wood’s surface roughness increased, the K value consequently increased in the aquades treatment. Prior studies [3,4] reported that rougher surfaces tended to result in lower *θ*_e_ and higher *K* values. Liquid permeated and diffused more extensively across the wood samples as surface roughness increased [11]. Furthermore, according to de Moura and Hernández (2006) [42], surfaces that have been sanded provide optimal circumstances for the spreading of liquids due to the presence of scratches caused by the abrasive grains. These scratches enhance the conduction of liquids through capillarity.

### 3.3. Wood–Adhesive Interphase Analysis

**Figure 7:**
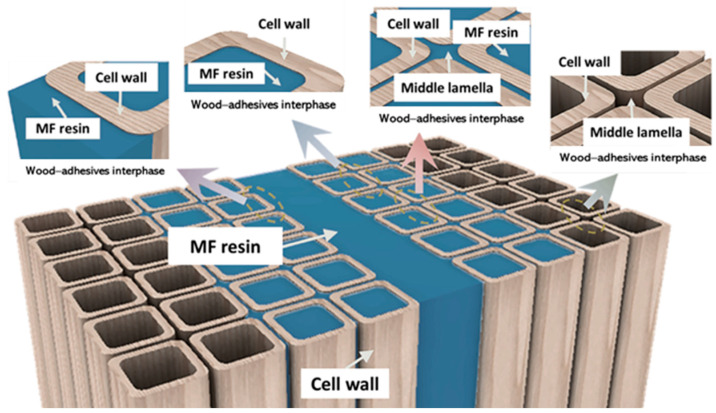
Illustration of woodadhesives interphase of melamine formaldehyde (MF) resins on Jabon wood.

The analysis of a wood–adhesive interphase entails the examination of the boundary between the wood surface and the adhesive substance. The wetting process of an adhesive on a solid surface has three main steps [55]: the establishment of interfacial adhesion at the surface; the spreading of an adhesive as a liquid flow over a solid surface; and the infiltration of a liquid into the inner regions of a porous solid. The interphase is essential in determining the overall performance and longevity of the adhesive bond. Several analytical techniques can be utilized for this objective, and DMA can be employed to investigate the viscoelastic characteristics of the interface between the adhesive and wood in question [1,38]. The interphase region exhibits an irregular or diverse layer [56]. Figure 7 displays the illustration of the wood–adhesive interphase of MF resins on Jabon wood. The interphase, within the context of wood bonding, is important in determining the strength and durability of the adhesive bond. In addition, according to [56], an adhesive’s bonding performance with wood elements is substantially impacted by the degree of the adhesive’s penetration into the porous network of interconnected layers.

**Figure 8:**
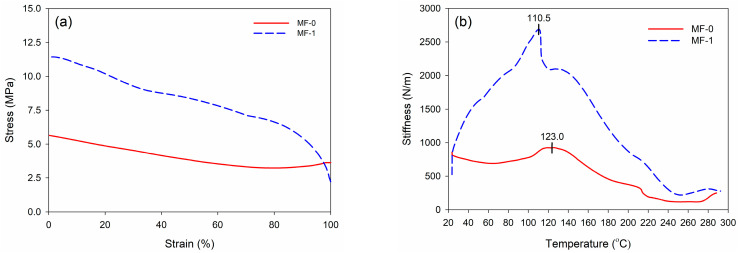
DMA results of wood–MF resin adhesive: (**a**) stress–strain curve and (**b**) stiffness as a function of temperature.

DMA involves the application of varying levels of stress to a material and the subsequent measurement of the resulting strain. Stress refers to the amount of force exerted on a certain region, whereas strain represents the extent of deformation or change in length compared to a material’s initial length. Figure 8a shows that MF-1 had a greater stress–strain curve compared to MF-0. A greater stress–strain curve suggests more stiffness in a material, indicating that the material is more resistant to deformation when subjected to dynamic loading. This result is in line with the stiffness of wood–MF resin as a function of temperature (Figure 8b). Wood–MF-0 reached a maximum stiffness of 957 N/m at 123.0 °C, while the wood–MF-1 had a maximum stiffness of 2734 N/m at 110.5 °C. The stiffness of wood adhesives is crucial in determining the overall structural integrity and performance of bonded wood products [57]. This study showed that MF-1 had a greater stiffness and could produce a better performance in its bonded wood products compared to MF-0.

**Figure 9:**
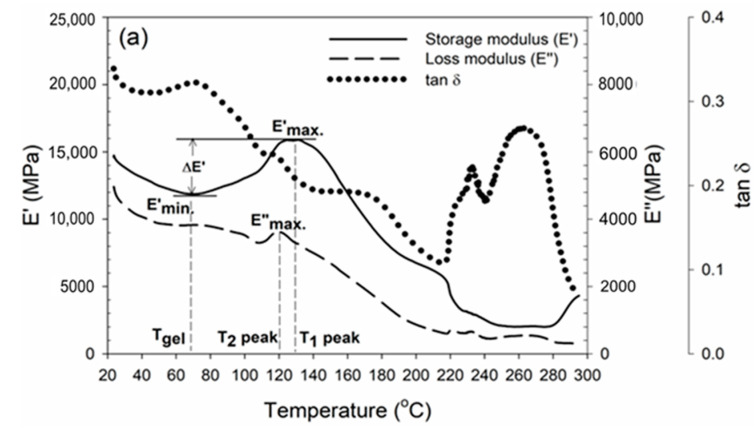

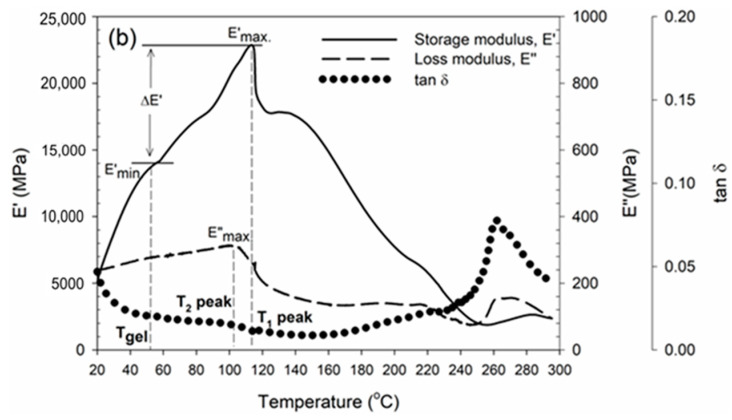
DMA thermograms of wood–MF resin adhesive: (**a**) MF-0 and (**b**) MF-1.

In line with the above results, the stiffness of wood adhesives is commonly quantified by the storage modulus (E’), which assesses an adhesive’s capacity to retain elastic energy. The storage modulus quantifies the inherent stiffness or rigidity of a material. Figure 9 displays the DMA thermograms of the wood–MF resin adhesives. Wood–MF-0 had a maximum storage modulus (E’_max_) of 12,650 MPa at a temperature of 128.9 °C, while wood–MF-1 had a maximum storage modulus (E’_max_) of 22,950 MPa at a temperature of 113.5 °C. The results showed that MF-1 had a greater storage modulus at a lower temperature compared to MF-0. In contrast, the loss modulus of the wood–MF-0 adhesive was higher than that of wood–MF-1. The loss modulus of a wood–adhesive interphase pertains to the capacity of the interface between the wood and the adhesive to release energy when subjected to dynamic loading conditions [57]. Various factors, such as adhesive formulation, curing conditions, wood surface preparation, and unique wood features, affect the loss modulus of the wood–adhesive interphase. The interface between wood and an adhesive is pivotal for the overall efficacy of wood–adhesive bonding as it influences parameters such as bond strength, endurance, and resilience to external conditions. Like the loss modulus, the tangent delta (tanδ) is a metric that quantifies the extent of damping or energy dissipation in a substance. It is commonly employed to describe the viscoelastic properties of a system. Tan δ in the wood–adhesive interphase context indicates the proportion of a material’s viscous (dissipative) reaction to its elastic (storage) response when subjected to dynamic loading circumstances.

**Figure 10:**
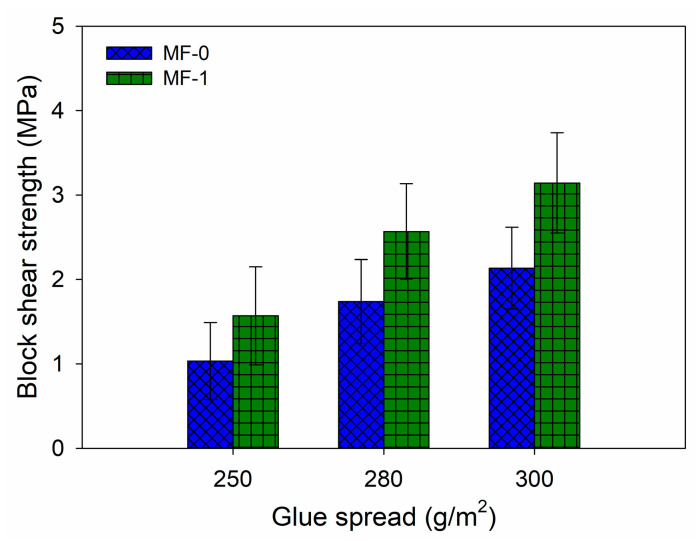
Bonding strength of MF resin adhesive at different amounts of glue spread.

The block shear strength results revealed that the adhesive strength of MF-0 with glue spreads of 250 g/m^2^, 280 g/m^2^, and 300 g/m^2^ was determined to be 1.03 MPa, 1.94 MPa, and 2.13 MPa, respectively (Figure 10). The shear strength values of the MF-0 samples were 30% lower than those of the MF-1 samples. The maximum block shear strength of MF-1 was 3.14 MPa, which was obtained with a glue application rate of 300 g/m^2^. This adhesive achieved bonding strengths of 1.56 MPa and 2.57 MPa when applied at glue spreads of 250 g/m^2^ and 280 g/m^2^, respectively. These results were in accordance with the results of the DMA (Figure 9), revealing that MF-1 has a greater storage modulus compared to MF-0.

## 4. Conclusions

The surface roughness and dynamic wettability of modified melamine formaldehyde-based adhesive on Jabon wood was investigated. Jabon wood treated with a P-100-grit sander had Ra values of 5.62 µm on the tangential surface (T), 5.77 µm on the radial surface (R), and 6.94 µm on the semi-radial surface (T/R). The tangential surface had a lower Ra value than the radial or semi-radial surfaces, indicating that the roughness level of Jabon wood on the tangential surface was preferable to those on the radial and semi-radial surfaces. The MF-based adhesives exhibited higher initial contact angles and equilibrium contact angles than the aquades. However, the speed of acquiring the equilibrium contact angle for the MF-based adhesives tended to be faster than that of aquades. The contact angle of the modified MF (MF-1) exceeded that of MF-0. On the other hand, the MF-0 adhesive exhibited a higher *K* value compared to the MF-1 adhesive, indicating that the MF-0 adhesive wets the surface of Jabon wood more easily than the MF-1 adhesive. The viscosity factor restricted the absorption of the MF-1 adhesive into the wood surface, resulting in a higher contact angle of MF-1 compared to MF-0. There are other factors, in addition to the contact angle and the wettability of the wood surface, that impact the performance of wood bonding when a particular type of adhesive is utilized. MF-1 exhibited a higher stress–strain curve and stiffness than MF-0, suggesting that it offers superior performance for bonded wood products and greater resistance to deformation under dynamic loading circumstances compared to MF-0.

## Figures and Tables

**Table 1:** Basic properties of MF-based adhesives [1].

Type of Adhesive	Properties			
	Solids Content (%)	Gelation Time (min, T = 100 °C)	Viscosity (cPs, T = 25 °C)	pH
MF-0	51.89 ± 0.36	30.27 ± 0.15	484.51 ± 3.69	7.67 ± 0.58
MF-1	48.56 ± 0.08	6.90 ± 0.10	570.75 ± 14.80	6.67 ± 0.58

**Table 2:** Surface roughness of Jabon wood after being sanded with a 100-grit belt sander.

Roughness Parameter	Tangential Surface (T)	Radial Surface (R)	Semi-Radial Surface (T/R)
Ra (mm)	5.62 ± 1.54	5.77 ± 1.65	6.94 ± 1.63
Rq (mm)	7.27 ± 2.13	7.14 ± 2.13	8.68 ± 1.89
Rz (mm)	33.21 ± 9.52	32.09 ± 10.64	39.09 ± 7.07

**Table 3:** Non-linear fitting results for aquades and MF-based adhesives on Jabon wood surfaces.

Liquid	Surface Type	Time to Reach the Equilibrium Contact Angle	*θ*_i_	*θ*_e_	Contact Angle Reduction Ratio	Wettability	
		(s)	(°)	(°)	(%)	*K* Value	R^2^
Aquades	T	11.13	75.5	26.914	64.35	0.269	0.981
	R	8.46	84.77	26.790	68.40	0.385	0.998
	T/R	8.93	86.4	24.056	72.16	0.422	0.991
MF-0	T	5.25	92.10	49.902	45.82	0.331	0.875
	R	5.74	102.65	58.682	42.83	0.262	0.904
	T/R	5.95	108.06	52.752	51.18	0.320	0.930
MF-1	T	7.95	120.13	66.611	44.55	0.212	0.962
	R	7.80	112.99	65.087	42.40	0.203	0.956
	T/R	11.50	126.08	74.124	41.21	0.136	0.977

## Data Availability

The authors can verify that the data substantiating the conclusions of this study are accessible within the paper. The primary data that substantiate the conclusions of this investigation can be obtained from the relevant author upon reasonable inquiry.

## References

[1] Amin Y., Adji R.P., Lubis M.A.R., Nugroho N., Bahtiar E.T., Dwianto W., Karlinasari L. (2023). Effect of Glue Spread on Bonding Strength, Delamination, and Wood Failure of Jabon Wood-Based Cross-Laminated Timber Using Cold-Setting Melamine-Based Adhesive. Polymers.

[2] Bekhta P., Krystofiak T., Proszyk S., Lis B. (2018). Evaluation of Dynamic Contact Angle of Loose and Tight Sides of Thermally Compressed Birch Veneer. Drv. Ind..

[3] Martha R., Dirna F.C., Hasanusi A., Rahayu I.S., Darmawan W. (2020). Surface Free Energy of 10 Tropical Woods Species and Their Acrylic Paint Wettability. J. Adhes. Sci. Technol..

[4] Yu Q., Pan X., Yang Z., Zhang L., Cao J. (2023). Effects of the Surface Roughness of Six Wood Species for Furniture Production on the Wettability and Bonding Quality of Coating. Forests.

[5] Buyuksari U., Akbulut T., Guler C., As N. (2011). Wettability and Surface Roughness of Natural and Plantation-Grown Narrow-Leaved ASH (*Fraxinus Angustifolia* Vahl.) Wood. BioResources.

[6] Sinn G., Gindl M., Stanzl-Tschegg S. (2004). Changes in the Surface Properties of Wood Due to Sanding. Holzforschung.

[7] Hendarto B., Shayan E., Ozarska B. (2006). Analysis of Roughness of a Sanded Wood Surface. Int. J. Adv. Manuf. Technol..

[8] Cahyono T.D., Wahyudi I., Priadi T., Febrianto F., Ohorella S. (2017). Sudut Kontak Dan Keterbasahan Dinamis Kayu Samama Pada Berbagai Pengerjaan Kayu. J. Tek. Sipil ITB.

[9] Walinder M. (2000). Wettability Phenomena on Wood: Factors Influencing Measurements Of Wood Wettability. Ph.D. Thesis.

[10] Shi S.Q., Gardner D.J. (2001). Dynamic Adhesive Wettability of Wood. Wood Fiber Sci..

[11] Darmawan W., Nandika D., Noviyanti E., Alipraja I., Lumongga D., Gardner D., Gérardin P. (2018). Wettability and Bonding Quality of Exterior Coatings on Jabon and Sengon Wood Surfaces. J. Coat. Technol. Res..

[12] Mu Q., Zhang Q., Yu W., Su M., Cai Z., Cui K., Ye Y., Liu X., Deng L., Chen B. (2020). Robust Multiscale-Oriented Thermoresponsive Fibrous Hydrogels with Rapid Self-Recovery and Ultrafast Response Underwater. ACS Appl. Mater. Interfaces.

[13] Mu Q., Cui K., Wang Z.J., Matsuda T., Cui W., Kato H., Namiki S., Yamazaki T., Frauenlob M., Nonoyama T. (2022). Force-Triggered Rapid Microstructure Growth on Hydrogel Surface for on-Demand Functions. Nat. Commun..

[14] Qin Z., Gao Q., Zhang S., Li J. (2014). Surface Free Energy and Dynamic Wettability of Differently Machined Poplar Woods. Bio. Res..

[15] Karlinasari L., Lestari A.T., Priadi T. (2018). Evaluation of Surface Roughness and Wettability of Heat-Treated, Fast-Growing Tropical Wood Species Sengon (*Paraserianthes Falcataria* (L.) I.C.Nielsen), Jabon (*Anthocephalus Cadamba* (Roxb.) Miq), and Acacia (*Acacia Mangium* Willd.). Int. Wood Prod. J..

[16] Lestari A.T., Wahyuningsih E., Syaputra M., Anwar H., Suparyana P.K., Ritonga F.N. (2021). Wettability and Treatability of Sengon (*Paraserianthes Falcataria* (L.) I.C. Nielsen) Wood from NTB. IOP Conf. Ser. Earth Environ. Sci..

[17] Vaziri M., Karlsson O., Abrahamsson L., Lin C.F., Sandberg D. (2021). Wettability of Welded Wood-Joints Investigated by the Wilhelmy Method: Part 1. Determination of Apparent Contact Angles, Swelling, and Water Sorption. Holzforschung.

[18] Huang Y., Qi Y., Zhang Y., Zhu R., Zhang Y., Yu W. (2019). Surface Properties of Novel Wood-Based Reinforced Composites Manufactured from Crushed Veneers and Phenolic Resins. Maderas Cienc. Y Tecnol..

[19] Ashori A. (2015). Effects of Extractives Removal on the Performance of Clear Varnish Coatings on Boards. J. Compos. Mater..

[20] Kúdela J. (2014). Wetting of Wood Surface by a Liquids of a Different Polarity. Wood Res..

[21] Santoni I., Pizzo B. (2011). International Journal of Adhesion & Adhesives Effect of Surface Conditions Related to Machining and Air Exposure on Wettability of Different Mediterranean Wood Species. Int. J. Adhes. Adhes..

[22] Gardner D.J., Generalla N.C., Gunnells D.W., Wolcott M.P. (1991). Dynamic Wettability of Wood. Langmuir.

[23] Wang S., Zhang Y., Xing C., Trocknungsverfahrens E. (2007). Effect of Drying Method on the Surface Wettability of Wood Strands. Holz. Roh. Werkst.

[24] Yuan Y., Lee T.R. (2013). Contact Angle and Wetting Properties.

[25] Peng Z., Jiang X., Si C., Joao Cárdenas-Oscanoa A., Huang C. (2023). Advances of Modified Lignin as Substitute to Develop Lignin-Based Phenol-Formaldehyde Resin Adhesives. ChemSusChem.

[26] Wiesner F., Klippel M., Dagenais C., Dunn A., Östman B., Janssens M.L., Kagiya K. Requirements for Engineered Wood Products and Their Influence on the Structural Fire Performance. Proceedings of the WCTE 2018 World Conference on Timber Engineering.

[27] Flatscher R.B.G., Schickhofer A.R.G. (2016). Cross Laminated Timber (CLT): Overview and Development. Eur. J. Wood Wood Prod..

[28] Li M., Zhang S., Gong Y., Tian Z., Ren H. (2021). Gluing Techniques on Bond Performance and Mechanical Properties of Cross-Laminated Timber (Clt) Made from Larix Kaempferi. Polymers.

[29] Lan P., Yang R., Mao H.Y., Cui J.Q., Brosse N. (2019). Production of Melamine Formaldehyde Resins Used in Impregnation by Incorporation of Ethylene Glycol and Caprolactam with High Flexibility, Storage Stability, and Low Formaldehyde Content. BioResources.

[30] Frihart C.R. (2015). Introduction to Special Issue Wood Adhesives: Past, Present, and Future. For. Prod. J..

[31] Santos J., Pereira J., Paiva N., Ferra J., Magalhães F.D., Martins J.M., de Carvalho L.H. (2021). Impact of Condensation Degree of Melamine-Formaldehyde Resins on Their Curing Behavior and on the Final Properties of High-Pressure Laminates. Proc. Inst. Mech. Eng. Part C J. Mech. Eng. Sci..

[32] Yuningsih I., Rahayu I.S., Lumongga D. (2019). Wettability and Adherence of Acrylic Paints on Long and Short Rotation Teaks. Wood Mater. Sci. Eng..

[33] Hartono R., Iswanto A.H., Herawati E., Suramana R.E., Sutiawan J., Amin Y., Sumardi I. (2022). The Improvement of Sumatran Elephant (Elephas Maximus Sumatranus) Dung Particleboard Characteristics Using Bamboo Layering. Polymers.

[34] Stalder A.F., Melchior T., Müller M., Sage D., Blu T., Unser M. (2010). Low-Bond Axisymmetric Drop Shape Analysis for Surface Tension and Contact Angle Measurements of Sessile Drops. Colloids Surfaces A Physicochem. Eng. Asp..

[35] Mu Q., Zhang Q., Gao L., Chu Z., Cai Z., Zhang X., Wang K., Wei Y. (2017). Structural Evolution and Formation Mechanism of the Soft Colloidal Arrays in the Core of PAAm Nanofibers by Electrospun Packing. Langmuir.

[36] Mu Q., Hu J. (2023). Polymer Mechanochemistry: From Single Molecule to Bulk Material. Phys. Chem. Chem. Phys..

[37] Chen B., Zhang S., Zhang Q., Mu Q., Deng L., Chen L., Wei Y., Tao L., Zhang X., Wang K. (2015). Microorganism Inspired Hydrogels: Fermentation Capacity, Gelation Process and Pore-Forming Mechanism under Temperature Stimulus. RSC Adv..

[38] Lubis M.A.R., Labib A., Sudarmanto, Akbar F., Nuryawan A., Antov P., Kristak L., Papadopoulos A.N. (2022). Influence of Lignin Content and Pressing Time on Plywood Properties Bonded with Cold-Setting Adhesive Based on Poly (Vinyl Alcohol), Lignin, and Hexamine. Polymers.

[39] Hamzah N.A., Razak N.A.A., Karim M.S.A., Salleh S.Z. (2022). Validation of a Roughness Parameters for Defining Surface Roughness of Prosthetic Polyethylene Pe-Lite Liner. Sci. Rep..

[40] Candan Z., Büyüksarı U., Korkut S., Unsal O., Nevzat C. (2012). Wettability and Surface Roughness of Thermally Modified Plywood Panels. Ind. Crops Prod..

[41] Sulaiman O., Hashim R., Subari K., Liang C.K. (2009). Effect of Sanding on Surface Roughness of Rubberwood. J. Mater. Process. Technol..

[42] De Moura L.F., Hernández R.E. (2006). Evaluation of Varnish Coating Performance for Three Surfacing Methods on Sugar Maple Wood. For. Prod. J..

[43] Zhong Z.W., Hiziroglu S., Chan C.T.M. (2013). Measurement of the Surface Roughness of Wood Based Materials Used in Furniture Manufacture. Meas. J. Int. Meas. Confed..

[44] Ugulino B., Hernández R.E. (2018). Analysis of Sanding Parameters on Surface Properties and Coating Performance of Red Oak Wood. Wood Mater. Sci. Eng..

[45] Cool J., Hernández R.E. (2011). Improving the Sanding Process of Black Spruce Wood for Surface Quality and Water-Based Coating Adhesion. For. Prod. J..

[46] Dai Q., Li M., Khonsari M.M., Huang W., Wang X. (2019). The Thermocapillary Migration on Rough Surfaces. Lubr. Sci..

[47] Rezaee Niaraki P., Krause A. (2020). Correlation between Physical Bonding and Mechanical Properties of Wood Plastic Composites: Part 1: Interaction of Chemical and Mechanical Treatments on Physical Properties. J. Adhes. Sci. Technol..

[48] Von Fraunhofer J.A. (2012). Adhesion and Cohesion. Int. J. Dent..

[49] Karlinasari L., Adzkia U., Sudarsono A.S., Larasatie P., Amin Y., Nugroho N. (2021). Surface Characteristics and Acoustical Properties of Bamboo Particle Board Coated with Polyurethane Varnish. Forests.

[50] Piao C., Winandy J.E., Shupe T.F. (2010). From Hydrophilicity To Hydrophobicity: A Critical Review: Part I. Wettability and Surface Behavior. Wood Fiber Sci..

[51] Whitesides G.M., Biebuyck H.A., Folkers J.P., Prime K.L. (1991). Acid-Base Interactions in Wetting. J. Adhes. Sci. Technol..

[52] Basri E., Martha R., Damayanti R., Rahayu I., Darmawan W., Gérardin P. (2022). Durability and Wettability of Varnishes on the Modified and Aged Surfaces of Short Rotation Teak Wood. Pigment Resin Technol..

[53] Gavrilovic-Grmusa I., Dunky M., Miljkovic J., Djiporovic-Momcilovic M. (2012). Influence of the Viscosity of UF Resins on the Radial and Tangential Penetration into Poplar Wood and on the Shear Strength of Adhesive Joints. Holzforschung.

[54] Darmawan W., Ginting M.B. (2020). Influence of Surface Roughness of Ten Tropical Woods Species on Their Surface Free Energy, Varnishes Wettability and Bonding Quality. Pigment. Resin Technol..

[55] Kim M., Park B.D. (2022). Effects of Molecular Weight of Urea–Formaldehyde Resins on Wettability and Adhesion at Wood Surface, Interphase, and Plywood. Wood Sci. Technol..

[56] Ülker O. (2016). Wood Adhesives and Bonding Theory. Adhesives—Applications and Properties.

[57] Karliati T., Lubis M.A.R., Dungani R., Maulani R.R., Hadiyane A., Rumidatul A., Antov P., Savov V., Lee S.H. (2024). Performance of Particleboard Made of Agroforestry Residues Bonded with Thermosetting Adhesive Derived from Waste Styrofoam. Polymers.

